# Estimation of Biochemical Pigment Content in Poplar Leaves Using Proximal Multispectral Imaging and Regression Modeling Combined with Feature Selection

**DOI:** 10.3390/s24010217

**Published:** 2023-12-30

**Authors:** Changsai Zhang, Yong Xue

**Affiliations:** 1 School of Environment and Spatial Informatics, China University of Mining and Technology, Xuzhou 221116, China; 2 School of Computing and Mathematics, College of Science and Engineering, University of Derby, Kedleston Road, Derby DE22 1GB, UK

**Keywords:** multispectral, plant physiological and biochemical parameters, sequential forward selection algorithm, support vector machine regression

## Abstract

Monitoring the biochemical pigment contents in individual plants is crucial for assessing their health statuses and physiological states. Fast, low-cost measurements of plants’ biochemical traits have become feasible due to advances in multispectral imaging sensors in recent years. This study evaluated the field application of proximal multispectral imaging combined with feature selection and regressive analysis to estimate the biochemical pigment contents of poplar leaves. The combination of 6 spectral bands and 26 vegetation indices (VIs) derived from the multispectral bands was taken as the group of initial variables for regression modeling. Three variable selection algorithms, including the forward selection algorithm with correlation analysis (CORR), recursive feature elimination algorithm (RFE), and sequential forward selection algorithm (SFS), were explored as candidate methods for screening combinations of input variables from the 32 spectral-derived initial variables. Partial least square regression (PLSR) and nonlinear support vector machine regression (SVR) were both applied to estimate total chlorophyll content (Chl_a+b_) and carotenoid content (Car) at the leaf scale. The results show that the nonlinear SVR prediction model based on optimal variable combinations, selected by SFS using multiple scatter correction (MSC) preprocessing data, achieved the best estimation accuracy and stable prediction performance for the leaf pigment content. The Chl_a+b_ and Car models developed using the optimal model had R^2^ and RMSE predictive statistics of 0.849 and 0.825 and 5.116 and 0.869, respectively. This study demonstrates the advantages of using a nonlinear SVR model combined with SFS variable selection to obtain a more reliable estimation model for leaf biochemical pigment content.

## 1. Introduction

Plant biochemical pigment contents, including chlorophyll and carotenoid contents, are crucial bio-indicators of plant physiology and functional processes up to the forest ecosystem level, including the light-harvesting reactions of photosynthesis, stress avoidance, and defense [1,2]. The accurate measurement of total chlorophyll content (Chl_a+b_) and carotenoid content (Car) at the leaf scale is of great importance for stress detection, growth status diagnosis, and studying the mechanisms of interaction between plants and the environment [3,4,5,6]. In addition, the biochemical pigment contents can affect the photosynthesis potential and growth rate of plants, directly relating to primary production and biomass accumulation [7]. As one of the most important forest tree species, poplar trees are widely cultivated for their economic and ecological benefits [8]. Quantifying photosynthetic activity at the leaf scale can provide important information on the growth status of poplar trees and further provide a scientific basis for the rational utilization and protection of forest resources [9,10].

The unique absorption characteristics of chlorophyll and carotenoid in the visible range make it possible to estimate their contents with spectroscopic techniques using hyper- or multi-spectral data [11]. In the early stages of estimation of non-destructive plant biochemical traits, non-imaging spectrometer technology was the main approach for in situ biochemical monitoring [12]. However, due to the inconsistency between the positions of sampling acquisition and spectral measurement on plant leaf surfaces based on five-point sampling, the non-imaging spectrometer was unable to accurately describe the distribution of biochemical content on the leaf surface. In recent years, hyperspectral spectroscopy imaging has been widely used for its ability to provide highly relevant and detailed spectral information on biochemical properties [13,14,15,16]. However, hyperspectral data suffer from data redundancy and band autocorrelation, termed “high dimensional disaster” [12]. Multispectral imaging sensors typically consist of four to a dozen broadband wavelength channels. For applications that do not require the whole spectral range in visible light or near-infrared bands (VIS-NIR), the detection performance of a multispectral sensor with tailor-made bands associated with the target property is as good as that of a hyperspectral sensor [17,18]. Additionally, multispectral imaging devices are more cost-effective. Therefore, multispectral imaging technology is better suited for monitoring biochemical traits to meet the need for a method with low cost and rapid response.

Due to the flexibility and feasibility of portable multispectral imaging devices, it is becoming more common for the scientific community to obtain proximal remote sensing data. Proximal remote sensing provides sub-millimeter or millimeter spatial resolution and higher temporal frequency than aerial and satellite data, which is particularly interesting for forestry management and precision agriculture [19,20,21,22]. By capturing images in close proximity to the plants, portable multispectral imaging devices enable more accurate monitoring of the physiological status and stress-related responses. Multiple spectral bands, or a single established vegetation index, contain relatively little information; thus, several studies have modeled the inversion of biochemical parameters by whole spectral bands or the combination of multiple vegetation indices derived from proximal multispectral images. Pan [23] extracted leaf chlorophyll concentration from nineteen-band multispectral images by utilizing PLSR analysis. The successive projections algorithm was implemented to determine optimal variables from whole spectral bands. Chungcharoen [24] estimated leaf SPAD using five-band multispectral images accompanied by four inversion modeling. The stepwise multiple linear regression was applied to variable selection for forty-four candidate variables derived from the multispectral images. Although the combination of spectral bands and vegetation indices can provide enhance spectral information, which is particularly valuable for multispectral imaging, the determination of sensitive spectral bands and indices is still a disturbing issue.

For the estimation of biochemical parameters, the combinations of spectral bands or indices used in most of the studies generally included a limited number of individual vegetation indices [13,19,25]. However, discrepancies exist in the selection of spectral bands or indices for modeling. Influencing factors such as optical spectrum-dividing mode, spectral bandwidth, signal-to-noise ratio, imaging distance, and lighting conditions contribute to variations in sensitive spectral bands [20]. Consequently, directly applying sensitive variables taken from different spectroscopy data for modeling with specific spectral data may not achieve optimal estimation accuracy in practice. In addition, although several studies have indicated that proximal multispectral imaging is a promising technique for quantifying biochemical traits [18,23,24], its potential is not fully realized due to the inadequacy of spectral information recorded by multispectral channels with broadband. To enhance multispectral information, the pseudo-hyper-spectrum can be established by the combination of spectral bands and vegetation indices associated with biochemical pigment contents that are derived from the multispectral bands. Meanwhile, it is also necessary to select the relevant feature variables derived from multispectral bands for the modeling of biochemical parameters in order to improve multispectral detection capabilities.

Feature variable selection can adaptively select the optimal subset to match target variables, reduce data dimensionality, and improve modeling accuracy and generalization [26]. At present, feature variable selection algorithms can be divided into three categories: filter, wrapper, and embedded [27,28,29]. Variable selection based on the filtering algorithm is independent of the model-training process. The filtering algorithm uses some evaluation criteria to screen out highly relevant variables with target parameters, such as correlation coefficient, mutual information, information entropy, etc. The wrapper algorithm takes into account both the induction learner’s performance and the importance of the variables. The algorithm evaluates the merits of feature variables through the evaluation function of the induction learner, which can select “tailor-made” variables for each model. The generation procedure for finding the best input variable combination based on the wrapper includes forward or backward searches, recursive feature selection, randomized search, etc. For embedding algorithms, variable selection is embedded into the model-training process through methods such as random forest, lasso, etc. Whatever the models are, their success depends upon the quality of the dataset, the selection of the feature variables, and the availability of effective method validation [30].

The objectives of this study were to evaluate the field application of proximal multispectral images that came with regression models based on the combination of input variable selected by different variable selection algorithms in order to predict the biochemical pigment contents of poplar leaves. The specific objectives were: (1) to select the spectral bands and vegetation indices derived from the proximal multispectral reflectance which were relevant to leaf Chl_a+b_ and Car based on three variable selection algorithms (CORR, RFE, and SFS); (2) to compare the accuracy of two linear and non-linear regression models (PLSR and SVR) based on the input variable combinations obtained through the variable selections; and (3) to evaluate the stability of the models based on optimal variable combination via leaf Chl_a+b_ and Car mapping analysis. This study can provide a technical basis for monitoring leaf biochemical parameters using proximal multispectral imaging.

## 2. Materials and Methods

### 2.1. Data Acquisition

#### 2.1.1. Sample Collection and Image Acquisition

**Figure 1:**
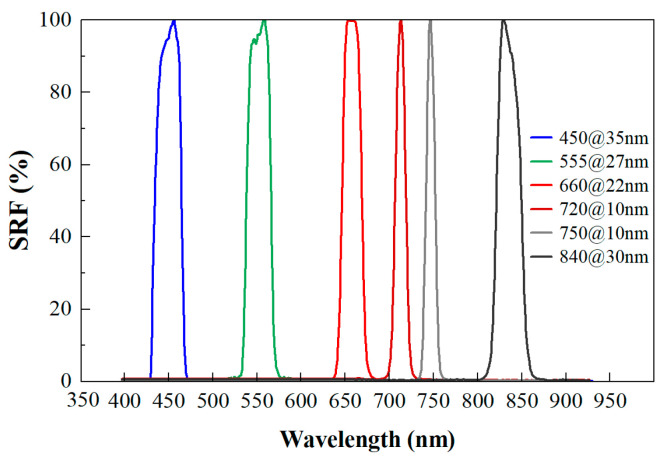
Spectral response curve (SRF) of MS600 multispectral instrument.

The samples were obtained from Pan’an Lake Wetland Park (34°22′33.7″ N; 117°23′21.8″ E, elevation 30 m a.s.l.), Xuzhou, China. To obtain the dataset of pigment contents with variations, the collection of poplar leaf samples was conducted based on the work of Shen Xin et al. [31], which considered the heterogeneity of biochemical contents in poplar leaves caused by differences in solar radiation in different positions in the canopy. In this study, the poplar leaves were divided into three layers according to height, and the leaf samples for the pigment content analyses were collected from the different height layers. For each layer of sampling, several mature leaves were randomly selected in various directions near the edge of the tree crown. The procedure for data acquisition and data analysis is shown in Figure 1.

**Figure 2:**
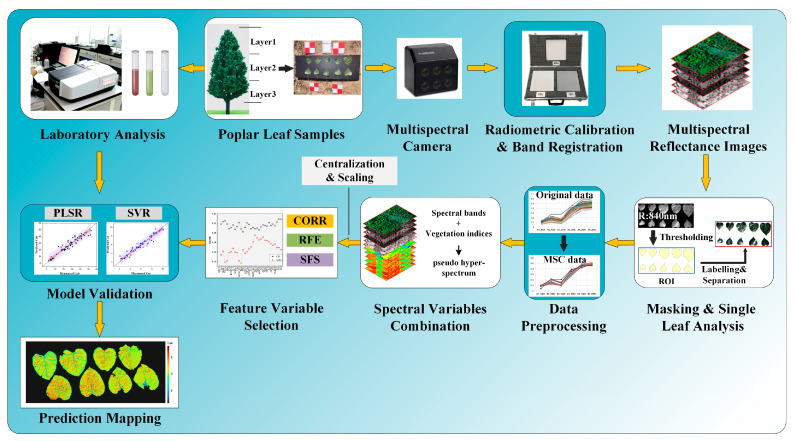
All key steps involved in processing proximal multispectral images for the purpose of acquiring leaf data, preprocessing images, building and selecting feature variables, validating the model, and predicting mapping at the leaf scale.

Using the MS600 multispectral instrument (Yusense, Inc., Qingdao, China), we obtained multispectral images of the leaves placed within the camera’s field of view. The multispectral instrument (MSI) contained six single-broadband channels with center wavelengths and spectral resolutions of 450@35 nm, 555@27 nm, 660@22 nm, 720@10 nm, 750@10 nm and 840@30 nm [32]. The corresponding spectral response curve is depicted in Figure 1. The field of view and pixel resolution were HFOV 49.5° × VFOV 38.1°, and 1280 × 960. The image bit depth was 16bit with the ‘tif’ storage format. Under sunny and cloud-free conditions, proximal multispectral images of 64 poplar leaves were collected outdoors from 11:00 to 13:00 on 17 October 2022. The leaf samples were tiled in batches according to layer height on a black background board. All multispectral images were captured at a height of 1.8 m by the camera. A reference whiteboard was positioned flat on the ground for each group image’s reflectance calibration. The overall technical processes are shown in Figure 2.

#### 2.1.2. Leaf Biochemical Measurements

The collected leaf samples were placed into an insulator before being sent for laboratory biochemical content analysis. The wet lab extraction technique was used to determine the chlorophyll and carotenoid concentrations per unit area of the leaves [33]. A leaf borer (diameter = 10 mm) was used to clip the fresh leaves. The pigment content for each leaf was extracted from 0.1g of fresh leaf with 10 mL 95% ethanol. After extraction, the absorbance of the extracts was measured with a UV–VIS spectrophotometer (MAPADA UV-1800PC, Shanghai, China), and the contents were determined and presented as the total chlorophyll content (Chl_a+b_) and carotenoid content (Car). The absorbance (A) of the samples was measured at 665 nm (for chlorophyll a), 649 nm (for chlorophyll b), and 470 nm (for carotenoid) using the spectrophotometer [34].

(1) $$\mathrm{Chlorophyll}\;a\;(\mathrm{mg}/L)=13{.95A}_{665}-6{.88A}_{649},$$

(2) $$\mathrm{Chlorophyll}\;b\;(\mathrm{mg}/L)=24{.96A}_{649}-7{.32A}_{665},$$

(3) $$\mathrm{Total}\;\mathrm{Chlorophll}\;(\mathrm{mg}/L)=\mathrm{Chla}+\mathrm{Chlb}=18{.08A}_{649}+6{.63A}_{665},$$

(4) $$\mathrm{Total}\;\mathrm{Carotenoid}\;(\mathrm{mg}/L)=\frac{{1000A}_{470}-2.05\mathrm{Chla}\;-114.8\mathrm{Chlb}}{245}.$$

The units used for the chlorophyll and carotenoid were subsequently converted to μg/cm^2^ using data on the volume of leaf pigment extract and the leaf disc area.

(5) $${\mathrm{Pigment}\;\mathrm{content}\;(\mu g/\mathrm{cm}}^{2})=\frac{C\;(\mathrm{mg}/L)\;\times \;n\;\times \;V\;(\mathrm{mL})\;}{{\mathrm{leaf}\;\mathrm{area}\;(\mathrm{cm}}^{2})}.$$

where C represents total chlorophyll or carotenoid concentration. n and V are the ethanol volume concentration and volume, respectively. Only the total chlorophyll and carotenoid were used in this study.

### 2.2. Image Pre-Processing

The leaf reflectance was acquired by the flat field radiometric calibration method, and the process was completed using ENVI 5.3. software (Exelis Inc., Herndon, VA, USA). Since only pixels belonging to the plant leaf area contained useful information, leaves were segmented from the background by eliminating all non-vegetation spectra. Considering high reflection levels in the near-infrared range on plant leaves, segmentation was performed by calculating the reflectance value at 840 nm for each image. The reflectance threshold value was set to 0.25 for the 840 nm images, and the respective mask images were used to segment each image individually. Each leaf was labeled in the segmented images for further data analysis.

To reduce the effects of specular reflection and leaf inclination, the images were processed by multiple scatter correction (MSC) [35]. MSC can eliminate spectral differences caused by different scattering levels and correct the baseline shift issue of spectral data through the “ideal spectrum”, thereby enhancing the correlation between spectra and lab data [36]. In this study, the ideal spectrum was determined by calculating the spectral average value of all leaf pixels in each group image, aiming to minimize variations.

In this study, the ideal spectrum was determined by calculating the spectral average value of all leaf pixels in each group image, aiming to minimize variations.

Vegetation indices have been widely used in ecological research for estimating and monitoring plant biochemical parameters, especially for photosynthetic pigments including chlorophyll and carotenoid [30,37]. Using the spectral reflectance data, we calculated 26 published vegetation indices. The indices included most of the existing vegetation indices related to pigment contents that could be calculated by the six bands of MSI, and the majority of the indices included were developed at the leaf scale [3]. The vegetation index formulas which were applied are presented in Table 1. The initial set of variables was established by the combination of 6 spectral bands and 26 vegetation indices derived from the multispectral bands. With the significance of the vegetation indices, the pseudo-hyper-spectrum could be formed to provide sufficient spectral information for the modeling of leaf pigment content.

### 2.3. Variable Selection

The purpose of variable selection is to identify the most accurate target property with the fewest variables and to reduce the model’s computational cost. As mentioned above, the initial set of variables included 6 spectral bands and 26 vegetation indices. Due to data redundancy and variable autocorrelation, it was impractical to utilize all of these variables for prediction modeling. Therefore, variable selection was applied as an initial stage of prediction modeling. Before variable selection, all variables in the initial set were normalized to compensate for the scale variations. In this study, three variable selection algorithms were applied to select an optimum variable subset for prediction modeling. All three variable selection algorithms selected the optimal combination of input variables with respect to their respective evaluation functions and by using their respective search strategies.

#### 2.3.1. Forward Filtering Algorithm with Correlation Analysis (CORR)

For the variable selection, we generally adopted the ‘Top-N’ strategy for feature variables, which directly removes low-correlation and unrelated variables with target properties from an initial set of variables through an empirical threshold based on correlation analysis. However, the n-top variables may not be the optimum subset for modeling due to the variable autocorrelation. For the whole initial set of variables, we established how many variables would be the optimal choice for modeling. A proposed approach called CORR was used to determine the optimal combination of variables and their quantity by a specific criterion rather than empirical threshold values. The CORR adopted a forward search method, which selected the variables with high relevance to target parameters as the input and iteratively added variables according to the order of absolute values of the correlation coefficient until all variables had been traversed. Adding candidate variables to the sorting order can avoid the removal of highly relevant variables. In this study, the partial least square method (PLS) and a support vector machine with a radial basis kernel (SVM) were applied as induction learners, and their root mean square error was used as the criterion to be minimized. A grid search with 5-fold repetition cross-validation was employed to determine the optimal parameters for the induction learners at each loop of CORR.

#### 2.3.2. Sequential Forward Selection Algorithm (SFS)

The sequential forward selection algorithm extends the variable subset from an initial set of variables in each iteration with the variable that increases the model performance the most [29]. SFS starts with an empty subset and adds variables to the subset in order to select the input variable combination with the best merit value based on the evaluation function. This iterative process should be performed until either the max variable number is reached or the merit value of the variable combination in the current iteration is worse than the merit value of the best input variable combination in the previous iteration [53,54]. In this study, a linear model regression learner (LM) and a support vector machine with a radial basis kernel (SVM) were utilized as induction learners, and their root mean square error was used as the criterion to be minimized. Resampling techniques were utilized in each iteration of the procedure to stabilize the feature rankings. Here, 5-fold cross-validation repetitions were employed.

#### 2.3.3. Recursive Feature Elimination Algorithm (RFE)

The recursive feature elimination algorithm is an iterative algorithm that works backward from an initial set of variables based on variable importance ranking [28]. RFE starts with all variables and repeatedly constructs an induction learner to recursively eliminate unimportant variables. The variables are sorted based on the weight of the induction learner, and the variable with the lowest-ranking score is eliminated at each loop of RFE [55]. The purpose is to find the variable subset that has the best merit value based on the evaluation function of the induction learner. In this study, RFE utilized the same criterion and induction learner as SFS.

### 2.4. Prediction Model

#### 2.4.1. Partial Least Square Regression (PLSR)

PLSR is a linear nonparametric model used for constructing a predictive model when input variables are many and highly colinear [13]. Partial least square regression combines the characteristics of principal component analysis, canonical correlation analysis, and linear regression analysis in the modeling process. PLSR reduces predictors to a small set of independent latent factors, which serve as new predictors, and regresses the response variables on these new predictors [56,57]. To determine the number of factors used in the model, a grid search was applied to select the optimal parameter for this study.

#### 2.4.2. Support Vector Machine Regression (SVR)

SVR is a nonparametric model that does not contain assumptions about the data distribution [13]. The method mathematically transfers the regression problem into a feature space with higher dimensionality than the original data space to facilitate a linear solution to an otherwise non-linear problem [57,58]. In this study, we used the radial basis function kernel in combination with a grid search for the optimization of C and γ. In order to avoid overfitting, C was set to vary from 0.1 to 10, and was combined with γ from 0.005 to 1 in the grid search.

In this study, we investigated proximal multispectral imaging techniques for the detection of the biochemical parameters of poplar leaves using variable selection and regression analysis. Linear and nonlinear regression analyses were implemented using an optimal combination of spectral bands and indices as the independent variable, and the poplar leaf pigment content as the dependent variable. The variable selection and regression modeling were performed using R package ‘mlr3fselect’ and ‘caret’.

### 2.5. Model Validation

To test how accurately the models predicted the values of biochemical contents at the leaf scale, the coefficients of determination (R^2^) and root mean square error (RMSE) were selected to display the error in the predicted value of the leaf pigment contents. Leave-one-out cross-validation (LOOCV) was utilized to obtain the merits in this study. These metrics were calculated as follows:

(6) $$R^{2}=\frac{\sum_{i=1}^{n}{\left(y_{i}-Y_{i}\right)}^{2}}{\sqrt{\sum_{i=1}^{n}\left(y_{i}-\bar{y}\right)}\;\sqrt{\sum_{i=1}^{n}\left(Y_{i}-\bar{Y}\right)}},$$

(7) $$\mathrm{RMSE}=\sqrt{\frac{1}{n}\sum_{i=1}^{n}{\left(y_{i}-\bar{Y}\right)}^{2}}.$$

where *n* represents the number of samples and *y_i_* and *Y_i_* represent the ith measured and *i*th predicted value, respectively. $\bar{y}$ and $\bar{Y}$ represent the average measured and average predicted value, respectively.

## 3. Results

### 3.1. Statistical Analysis

**Figure 3:**
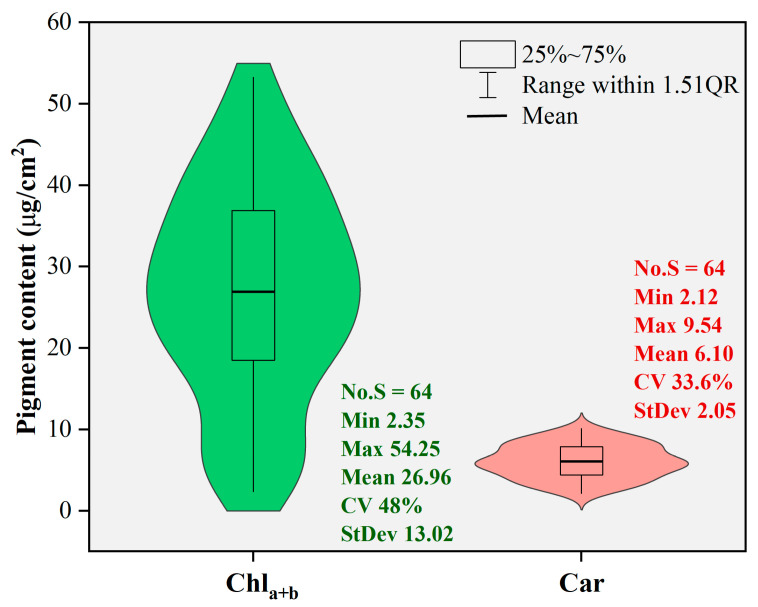
Descriptive statistical analysis of pigment content values.

Figure 3 shows the statistical characteristics of the leaf pigment content values obtained from the laboratory reference analysis. The analysis involved sixty-four poplar leaf samples. The range of leaf chlorophyll content (Chl_a+b_) values was 2.35–54.25, with a mean of 26.96 and a standard deviation of 13.02. For leaf carotenoid content (Car), the range was 2.12–9.54, with a mean of 6.10 and a standard deviation of 2.05. Both datasets appeared to have approximately normal distribution, with coefficients of variation of 48% for Chl_a+b_ and 33.6% for Car. The high variabilities of the pigment content levels were helpful for modeling purposes in this study.

**Figure 4:**
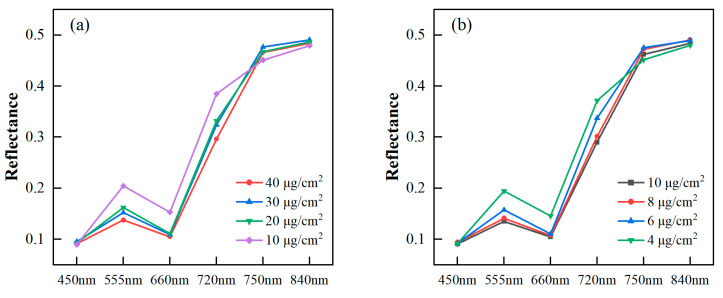
Spectral reflectance of poplar leaves with different Chl_a+b_ levels (**a**) and Car levels (**b**).

All samples were divided into four groups according to their pigment content levels. The average pigment content for each group was analyzed to observe changes in the image’s spectral reflectance with the varying content levels. In Figure 4, the multispectral reflectances of the six bands preprocessed by MSC are depicted in a polygonal map. The spectral reflectances of poplar leaves decreased in the green (555 nm), red (660 nm), and red edge (720 nm) bands as Chl_a+b_ and Car increased. The reason for this phenomenon was that the increased leaf Chl_a+b_ and Car level led to heightened absorption in the visible light region, resulting in decreased leaf reflectance. Previously, vegetation indices, specifically a green peak and red edge in the visible light region, were identified and utilized for Chl_a+b_ estimation [59]. For the blue (450 nm) band, there was almost no discrepancy in reflectance under different pigment content levels. The reason might be the low signal-to-noise ratio in the blue channels of the MSI. From the red edge (750 nm) to the near infrared (840 nm), there was no significant change in reflectance. This observation aligned with the fact that the leaf reflectance was not affected by photosynthetic pigments in the near infrared, maintaining a consistently high reflectance level [11].

**Figure 5:**
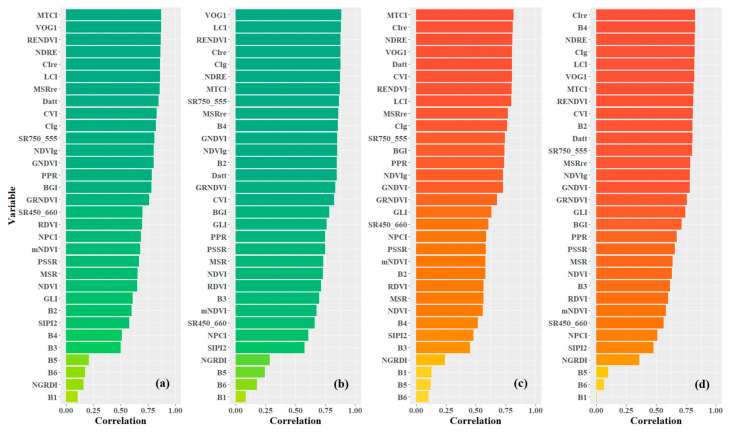
Pearson’s correlation ranks of variable candidates (**a**,**c**) are variables from the original data (OS); (**b**,**d**) are variables from the MSC preprocessing data. The figure expresses the correlation of variables with Chl_a+b_ using green bars and with Car using orange bars.

As mentioned in Section 2.2., the candidate variables for prediction modeling consisted of 32 variables, including 6 spectral bands and 26 vegetation indices. Figure 5 shows the ranks of variables based on the absolute value of Pearson’s correlation between the variables and the leaf pigment contents. Two types of variable data were considered, including no-preprocessing original data (OS) and preprocessing data using multiple scatter correction (MSC). According to the distribution of pigment content in poplar leaves, the variables with the highest correlation coefficient with Chl_a+b_ and Car for OS data were both MTCI, with values of 0.869 and 0.814, respectively. For MSC data, the variables with the highest coefficients with Chl_a+b_ and Car were VOG1 (0.884) and CIre (0.827), respectively. After MSC preprocessing, the number of variables with Pearson’s coefficients greater than 0.85 increased from 8 to 11 for Chl_a+b_, while the number of variables with Pearson’s coefficients greater than 0.80 increased from 7 to 13 for Car. These results indicate that MSC preprocessing can enhance the spectral variable information related to the leaf pigment contents of poplar leaves. Furthermore, the overall correlation between Chl_a+b_ and spectral variables was higher than that of Car. The spectral indices presented in this paper, typically used for Chl_a+b_ inversion [3], were found to be highly relevant to Car in this study. This was primarily due to the broadband spectral overlap of carotenoid and chlorophyll absorption. For the spectral indices’ formulas with high relevance to the leaf pigment contents, most of them were related to the responses of the green, red, and red edge bands.

### 3.2. Input Variable Selection

A total of 32 variable candidates, including 6 bands and 26 indices, were established to select the input variables combination suitable for estimating leaf pigment contents. Three variable selection algorithms were implemented in this paper: CORR, RFE, and SFS. The root mean square error (RMSE) of the induction learner was utilized as the evaluation function.

**Figure 6:**
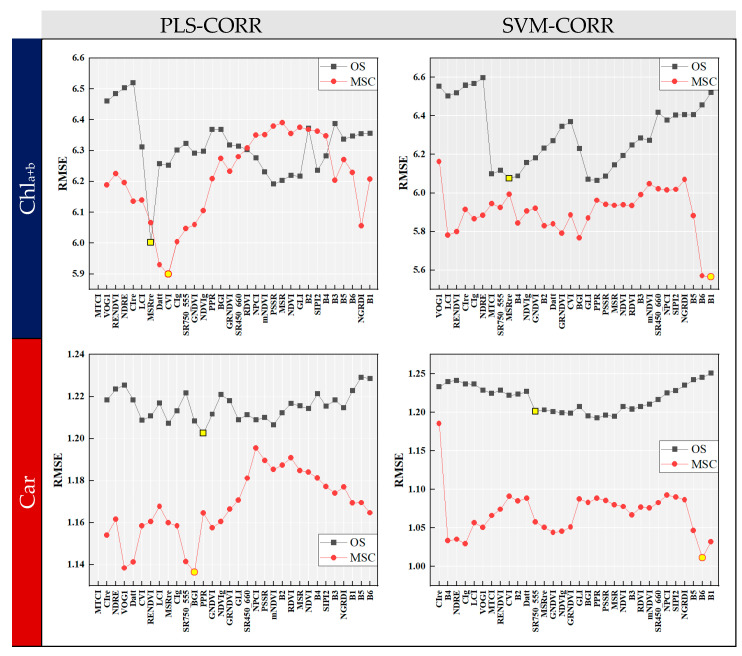
RMSE curves of forward selection algorithm with correlation analysis (CORR). The optimal combination of input variables for modeling could be determined by identifying the set of variables before the point (marked with yellow circles and squares) where the RMSE was the lowest.

Figure 6 shows the RMSE curves of the CORR algorithm combined with two induction learners. The variables at the x-axis were arranged in the same order as that depicted in Figure 4. The RMSE curve displayed fluctuations as the number of variables increased, and an overall decrease was observed in RMSE when using MSC data preprocessing. The optimal combination of input variables for modeling could be determined by identifying the set of variables before the point where the RMSE was the lowest. For OS data, PLS-CORR selected 6 and 12 input variables relevant to Chl_a+b_ and Car, respectively, while SVM-CORR selected 18 for both. With MSC preprocessing, PLS-CORR identified 8 and 11 optimal input variables for Chl_a+b_ and Car, while SVM-CORR selected 31 and 30, respectively. It is noteworthy that SVM-CORR identified a larger number of variables compared to PLS-CORR, suggesting that it was less effective in terms of reducing data dimensionality. Overall, the CORR algorithm can serve as a guide for feature variable selection when employing the ‘Top-N’ approach.

Unlike the CORR filtering algorithm, RFE and SFS are wrapper algorithm. They employed interior feature ranking to screen the optimal variables combination by determining induction learner performance. RFE and SFS removed the least important variable and recalculated the rankings for the remaining variables at each iteration until the optimal combination of variables was selected based on the evaluation function of the instruction learner. Table 2 presents the final variable selection results of different algorithms. For OS data, LM-RFE selected 5 and 4 variables for Chl_a+b_ and Car, while SVM-RFE selected 24 and 15, respectively. Using MSC data, LM-RFE selected 5 and 6 variables for Chl_a+b_ and Car, while SVM-RFE selected 15 and 25, respectively. For both data treatment types and for both leaf pigment contents, LM-SFS and SVM-SFS selected seven and three variables, respectively. The optimal variable combination selected by SVM-SFS included at least two variables with high correlation and one variable with low correlation. These results suggest that SFS, particularly SVM-based SFS, effectively reduces data dimensionality.

With the significance of the vegetation indices, the initial variable set, called the pseudo-hyper-spectrum, could be formed to enhance multispectral information. This initial variable set allowed for the selection and modeling of feature variables relevant to the target parameters, thereby improving multispectral detection capabilities. The results pointed out that the ability to reduce data dimensionality among various variable selection algorithms showed significant differences. The SFS was superior to CORR and RFE in this regard. Specifically, SVM-SFS identified an optimal variable combination from the initial set, retaining only three specified variables with sufficient significance to account for leaf Chl_a+b_ and Car.

### 3.3. Model Comparation

**Figure 7:**
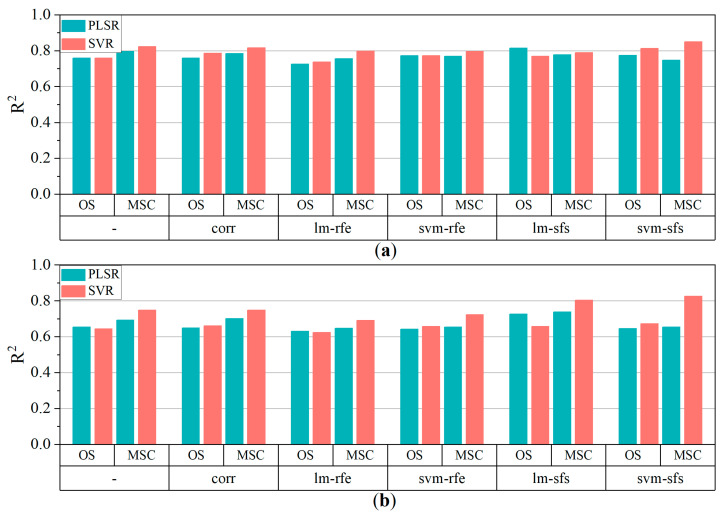
R^2^ of multiple models (**a**) Chl_a+b_, (**b**) Car. ‘-’ represents no variable selection. Whole candidate variables were utilized as input variables for modeling.

According to the variable selection process, an optimal variable combination can provide acceptable accuracy when an appropriate regression model is utilized. Linear PLSR and nonlinear SVR were both utilized to train the models to estimate the pigment contents of poplar leaves. Figure 7 depicts the R^2^ of model fits which was achieved suing the PLSR and SVR. In comparison to the results using the whole initial variable set from the OS and MSC data, CORR slightly improved the estimation accuracy, while the models combined with RFE exhibited the lowest accuracy for both pigment contents. This indicated that RFE was not suitable for selecting feature variables when only continuous variables were used in regression modeling. The model fit results revealed that lm-sfs and svm-sfs was the most effective variable selection algorithms in terms of improving estimation accuracy for OS and MSC data, respectively.

**Figure 8:**
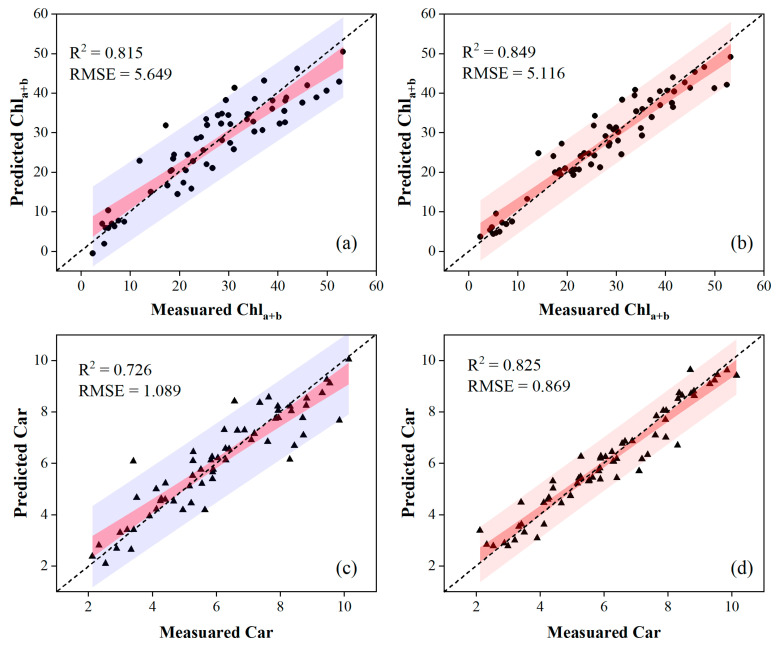
Scatter plots of the optimal regression models evaluating the pigment contents. The first column of the figure was obtained via the PLSR model using input variables selected by lm-sfs for OS data, and the second column was obtained via the nonlinear SVR model using input variables selected by svm-sfs for MSC data.

Table 3 and Table 4 present the accuracy of the validation results by comparing regression models combined with different variable selections. The PLSR combined with lm-sfs produced the highest estimation accuracy for leaf pigment contents on OS data, while the nonlinear SVR combined with svm-sfs outperformed other methods using MSC data. Specifically, the nonlinear SVR combined with svm-sfs, which used MSC data, provided a good estimation capability, yielding an R^2^ of 0.849 and an RMSE of 5.116 for Chl_a+b_, and an R^2^ of 0.825 and RMSE of 0.869 for Car. Another model, employing the PLSR combined with lm-sfs and OS data, estimated an R^2^ of 0.818 and an RMSE of 5.633 for Chl_a+b_, and an R^2^ of 0.726 and RMSE of 1.089 for Car, respectively. These results also indicated that the accuracy of pigment content estimates in poplar leaves could be improved through MSC data preprocessing. Scatter plots depicting the models with the highest estimation accuracies using OS and MSC data are presented in Figure 8.

### 3.4. Leaf Parameter Mapping

**Figure 9:**
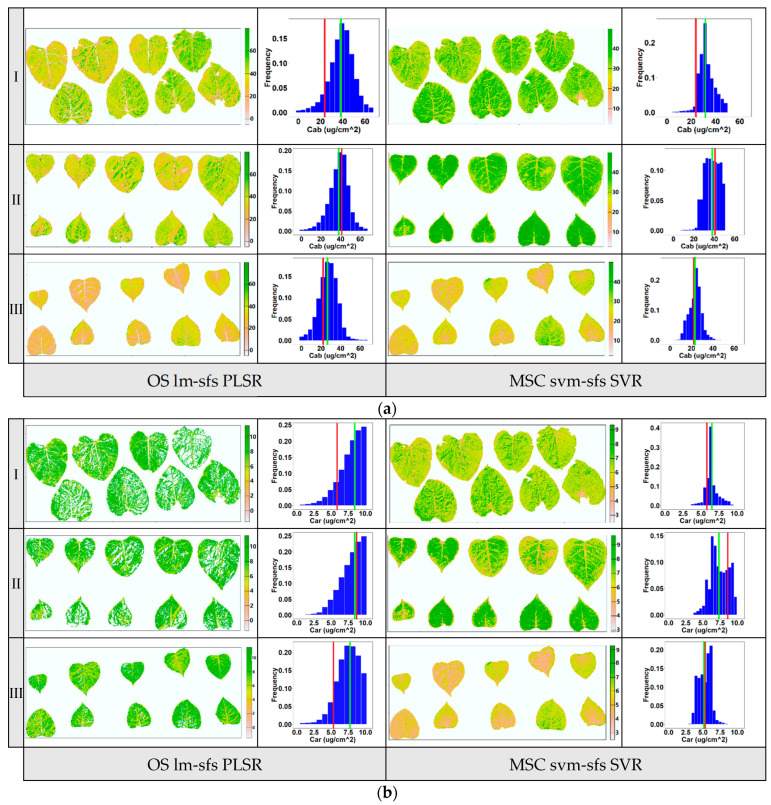
Visualization maps for Chl_a+b_ (**a**) and Car (**b**) distribution at the leaf scale in three vertical layers within a poplar canopy (I: upper canopy layer, II: middle canopy layer, III: lower canopy layer). The first column in the figure was obtained via the PLSR model using input variable selected by lm-sfs, and the second column was obtained via the nonlinear SVR model using input variable selected by svm-sfs. Red line in the frequency histogram: lab measurement value; green line: average predicted value of pixels.

To evaluate the stability of the models with the highest predictive ability, as mentioned above, the pigment contents of poplar leaf samples from three layers at vertical direction were visually mapped. The previous report indicated that the pigment content of poplar leaves varied depending on their location within the poplar canopy and was influenced by solar radiation conditions, with upper-canopy leaves generally having lower pigment contents than lower-canopy leaves [31]. In this study, the distribution of pigment contents in leaf samples from three layers within a poplar canopy (upper, middle, and lower layers) was mapped using the PLSR based on optimal variables selected by lm-sfs (Figure 9a) and the nonlinear SVR based on optimal variables selected by svm-sfs (Figure 9b). The levels of Chl_a+b_ and Car are depicted in different colors, ranging from high (green) to low (orange). The spatial t distribution of pigment contents in these leaves was visualized via pixel analysis of multispectral images. The frequency histogram illustrates the distribution of pigment contents for all pixels in each layer of the leaf samples. These results provide insights into the heterogeneity of pigment contents in poplar leaf samples across different layers. Additionally, the visualization results revealed significant differences in the distribution of pigment content between poplar leaves and veins, as well as notable variations in pigment distribution within some senescent leaves. This indicates that the distribution of pigment content on the surface of a single leaf exhibited spatial inconsistency.

As shown in the histogram maps (Figure 9), the average predicted value of image pixels and the corresponding lab measurement value of leaf samples at each layer are annotated with green and red lines, respectively. Compared to the PLSR model using OS data, the average predicted values derived from the nonlinear SVR model using MSC data were more consistent with the lab measurement values. Although the PLSR model achieved good estimation accuracy, as mentioned above, notable discrepancies existed between the average predicted value and lab measurement value of pigment content, particularly for the Car with lower variation coefficients (the white areas in the leaves in Figure 8b were removed due to outliers). The predicted value of PLSR using OS data showed a more dispersed distribution than that of SVR using MSC data, indicating that the predicted pigment contents had been overestimated. Overall, the results suggest that the nonlinear SVR model outperformed the PLSR model in terms of prediction accuracy and stability for the leaf pigment contents. The primary reason for this discrepancy was that PLSR linear regression was relatively sensitive to input parameters and achieved low estimation performance when the input variables had nonlinear distributions. Additionally, compared to SVR nonlinear regression, the PLSR linear regression model lacked the flexibility to capture the complex patterns of spectral reference in the leaf images [19]. The results demonstrate that the nonlinear mapping and generalization of nonlinear SVR were superior to that of PLSR. Thus, among the models developed for leaf pigment content in this study, the SVR combined with svm-sfs using MSC preprocessing data improved the performance of proximal multispectral imaging for the purpose of estimating the biochemical pigment contents of poplar at the leaf scale.

## 4. Conclusions

Using proximal multispectral images of poplar leaves as information with which to predict the biochemical pigment contents in the leaves via variable selection and regression analyses was the primary purpose of this work. A few main conclusions can be established from this study. (1) Reflectance correction used MSC preprocessing to allow for freedom from leaf architecture effects (specular reflection and leaf inclination) and to improve proximal imaging spectral information related to Chl_a+b_ and Car at the leaf scale. (2) SFS-SVR achieved a higher performance for the estimation of bio-parameters in terms of reducing data redundancy and enhancing estimation accuracy compared to the PLSR model combined with CORR and RFE. (3) The nonlinear mapping and generalization of proximal multispectral VIs-based modeling by SVR were superior to those of PLSR.

Finally, this study showed that proximal multispectral imaging combined with SFS variable selection and nonlinear SVR model is a promising technology for the monitoring of leaf biochemical pigment content. Its use can be expanded to plant phenology or ecology issues. Applying the optimal prediction model to whole multispectral images produces a map of spatial pigment contents. It will, thus, be possible to follow up on pigment content dynamics at each leaf level, contributing to improving our understanding of the heterogeneous distribution of physiological traits and the mechanisms underlying environmental stress responses. In addition, low-cost proximal multispectral imaging for biochemical monitoring can be used as an aspect of phenological observation, which is particularly valuable as it facilitates the need for the periodic acquisition of temporal data.

## Figures and Tables

**Table 1:** Summary of spectral parameters, wavebands, and citations for chlorophyll and carotenoid at the leaf scale.

Vegetation Index	Abbreviation	Formulation	Reference
Blue green pigment index	BGI	Blue/Green	[38]
Chlorophyll index using green reflectance	CI_green_	(NIR/Green) − 1	[39]
Chlorophyll index using red edge reflectance	CI_red-edge_	(NIR/Edge1) − 1	[39]
Chlorophyll vegetation index	CVI	NIR × (RED/$B{\mathrm{lue}}^{2}$)	[40]
Datt	Datt	(NIR − Edge1)/(NIR − Red)	[41]
Green leaf index	GLI	(2 × Green − Red − Blue)/(2 × Green + Red + Blue)	[42]
Green normalized difference vegetation index	GNDVI	(NIR − Green)/(NIR + Green)	[43]
Green-red NDVI	GRNDVI	(NIR − Green − Blue)/(NIR + Green + Blue)	[44]
Leaf chlorophyll index	LCI	(NIR − Edge1)/(NIR + Red)	[41]
Modified NDVI	mNDVI	(NIR − Red)/(NIR + Red − 2 × Blue)	[3]
Modified simple ratio	MSR	[(NIR/Red) − 1)]/($\sqrt{\mathrm{NIR}/\mathrm{Red}+1}$]	[45]
Modified red-edge simple ratio	MSR_red-edge_	(NIR − Edge1 − 1)/$(\sqrt{\mathrm{NIR}-\mathrm{Edge}1+1}\;$)	[45]
MERIS terrestrial chlorophyll index	MTCI	(Edge2 − Edge1)/(Edge1 − Red)	[46]
Normalized difference red edge index	NDRE	(NIR − Edge1)/(NIR + Edge1)	[47]
Normalized difference vegetation index	NDVI	(NIR − Red)/(NIR + Red)	[48]
Green NDVI	NDVIg	(Edge2 − Green)/(Edge2 + Green)	[43]
Normalized green-red difference index	NGRDI	(Green − Red)/(Green + Red)	[38]
Normalized pigment chlorophyll index	NPCI	(Red − Blue)/(Red + Blue)	[49]
Normalized difference vegetation index	PPR	(Green − Blue)/(Green + Blue)	[50]
Pigment specific simple ratio	PSSR	NIR/Red	[51]
Renormalized difference vegetation index	RDVI	(NIR − Red)/($\sqrt{\mathrm{NIR}+\mathrm{Red}}$)	[52]
Red edge normalized difference vegetation index	RENDVI	(Edge2 − Edge1)/(Edge2 + Edge1)	[43]
Structure insensitive pigment index 2	SIPI2	(NIR − Blue)/(NIR − Red)	[51]
Simple ratio vegetation index 450/660	SR_450/660_	Blue/Red	[49]
Simple ratio vegetation index 750/555	SR_750/555_	Edge2/Green	[39]
Vogelmann red edge index 1	VOG1	Edge2/Edge1	[49]

**Table 2:** The results of input variable selection based on different algorithms.

Type	Variable Selection Algorithm	Chl_a+b_ (μg/cm^2^)		Car (μg/cm^2^)	
		Number of Variables	Input Variables	Number of Variables	Input Variables
OS	pls-corr	6	MTCI VOG1 RENDVI NDRE CIre LCI	12	MTCI CIre NDRE VOG1 Datt CVI RENDVI LCI MSRre CIg SR750_555 BGI
	svm-corr	18	MTCI VOG1 RENDVI NDRE CIre LCI MSRre Datt CVI CIg SR750_555 NDVIg GNDVI PPR BGI GRNDVI SR450_660 RDVI	18	MTCI CIre NDRE VOG1 Datt CVI RENDVI LCI MSRre CIg SR750_555 BGI PPR NDVIg GNDVI GRNDVI GLI SR450_660
	lm-rfe	5	NDRE LCI CIre VOG1 NDVI	4	NDRE LCI CIre VOG1
	svm-rfe	24	RENDVI VOG1 MTCI NDRE CIre LCI MSRre Datt CIg CVI SR750_555 NDVIg GNDVI PPR BGI GRNDVI SIPI2 mNDVI PSSR RDVI MSR NDVI SR450_660 NPCI	15	RENDVI VOG1 MTCI NDRE CIre LCI Datt CVI CIg MSRre SR750_555 BGI PPR NDVIg GNDVI
	lm-sfs	10	B2 B4 B5 BGI GNDVI LCI MSRre MTCI PPR VOG1	20	B2 BGI CIg CIre GNDVI GRNDVI LCI MSR MSRre NDVI NDVIg NPCI PPR PSSR RDVI RENDVI SIPI2 SR450_660 SR750_555 VOG1
	svm-sfs	7	CIg CVI LCI MSRre RENDVI SR450_660 VOG1	7	BGI CIg GNDVI NDVIg PPR RENDVI SR450_660
MSC	pls-corr	8	VOG1 LCI RENDVI CIre CIg NDRE MTCI SR750_555	11	CIre B4 NDRE CIg LCI VOG1 MTCI RENDVI CVI B2 Datt
	svm-corr	31	CIre B4 NDRE CIg LCI VOG1 MTCI RENDVI CVI B2 Datt SR750_555 MSRre GNDVI NDVIg GRNDVI GLI BGI PPR PSSR MSR NDVI B3 RDVI mNDVI SR450_660 NPCI SIPI2 NGRDI B5 B6	30	CIre B4 NDRE CIg LCI VOG1 MTCI RENDVI CVI B2 Datt SR750_555 MSRre GNDVI NDVIg GRNDVI GLI BGI PPR PSSR MSR NDVI B3 RDVI mNDVI SR450_660 NPCI SIPI2 NGRDI B5
	lm-rfe	5	MSR NDVI PSSR LCI NDRE	6	MSR NDVI PSSR NDRE PPR mNDVI
	svm-rfe	15	VOG1 LCI RENDVI CIg GRNDVI NDRE CIre SR750_555 NDVIg GNDVI B2 MTCI MSRre B4 Datt	25	CIg RENDVI VOG1 LCI B2 B4 GRNDVI NDRE CIre NDVIg GNDVI SR750_555 MTCI CVI Datt MSRre BGI PPR GLI B6 NDVI PSSR MSR B3 RDVI
	lm-sfs	9	CIg Datt GNDVI MSR MTCI NDVIg NGRDI SR750_555 VOG1	10	B2 B5 B6 CIg CIre GLI MSRre MTCI NDRE RENDVI
	svm-sfs	3	B6 MTCI VOG1	3	B4 B6 RENDVI

**Table 3:** The validation accuracy of Chl_a+b_ model using LOOCV.

Type	Variable Selection Algorithm	PLSR		SVR	
		R^2^	RMSE	R^2^	RMSE
OS	-	0.760	6.452	0.759	6.480
	corr	0.759	6.454	0.787	6.089
	lm-rfe	0.726	6.884	0.737	6.728
	svm-rfe	0.772	6.304	0.773	6.280
	lm-sfs	**0.815**	**5.649**	0.769	6.352
	svm-sfs	0.774	6.247	0.813	5.694
MSC	-	0.796	6.207	0.823	5.566
	cor-fs	0.785	6.082	0.816	5.637
	lm-rfe	0.755	6.507	0.798	5.923
	svm-rfe	0.769	6.324	0.796	5.922
	lm-sfs	0.778	6.265	0.789	6.059
	svm-sfs	0.748	6.598	**0.849**	**5.116**

‘-’ represents no variable selection; whole candidate variables were utilized as input variables. Bold indicates the model which yielded the highest accuracy for two data types (OS and MSC).

**Table 4:** The validation results of Car model using LOOCV.

Type	Variable Selection Algorithm	PLSR		SVR	
		R^2^	RMSE	R^2^	RMSE
OS	-	0.654	1.229	0.645	1.251
	corr	0.649	1.224	0.661	1.204
	lm-rfe	0.630	1.258	0.624	1.268
	svm-rfe	0.642	1.237	0.657	1.212
	lm-sfs	**0.726**	**1.089**	0.657	1.215
	svm-sfs	0.646	1.232	0.673	1.185
MSC	-	0.693	1.175	0.748	1.037
	cor-fs	0.702	1.139	0.749	1.037
	lm-rfe	0.648	1.228	0.691	1.156
	svm-rfe	0.654	1.216	0.724	1.092
	lm-sfs	0.739	1.058	0.804	0.919
	svm-sfs	0.655	1.215	**0.825**	**0.869**

‘-’ represents no variable selection; whole candidate variables were utilized as input variables. Bold indicates the model which yielded the highest accuracy for two data types (OS and MSC).

## Data Availability

Data are contained within the article.
